# A Novel Chorioallantoic Membrane (CAM) Setup to Investigate Angiogenic Effects of Extracorporeal Shock Wave Therapy (ESWT)

**DOI:** 10.3390/cells15161471

**Published:** 2026-08-17

**Authors:** Lorenz Faihs, Jonas Flatscher, Cyrill Slezak, Bardia Firouz, Nassim Ghaffari Tabrizi-Wizsy, Kurt Schicho, Paul Slezak, Peter Dungel

**Affiliations:** 1 Ludwig Boltzmann Institute for Experimental and Clinical Traumatology, 1200 Vienna, Austria; lorenz.faihs@kh-herzjesu.at (L.F.); jonas.flatscher@lbg.ac.at (J.F.);; 2 Department of Orthopedics, Herz Jesu Hospital, 1030 Vienna, Austria; 3 Department of Physics, Utah Valley University, Orem, UT 84058, USA; 4 Department of Urology, Klinik Ottakring, 1160 Vienna, Austria; 5 SFL Chicken CAM Lab, Otto Loewi Research Center, Department of Immunology and Pathophysiology, Medical University of Graz, 8036 Graz, Austria; 6 Department of Oral and Maxillofacial Surgery, Medical University of Vienna, 1090 Vienna, Austria

**Keywords:** angiogenesis, chorioallantoic membrane, CAM, extracorporeal shock wave therapy, ESWT, blood-supply-related diseases

## Abstract

Extracorporeal shock wave therapy (ESWT) is a well-known biophysical therapy that offers several beneficial effects, including a presumed increase in the growth of blood vessels. The chorioallantoic membrane (CAM) assay is a well-established in vivo model for studying angiogenesis. The aim of this study was to develop an experimental setup to apply ESWT to CAM in a three-step process and investigate the vascular response. First, we conducted virtual simulations of various shock-wave application setups to CAM and subsequently tested them in preliminary studies to evaluate their feasibility. In the final stage, we employed the most suitable protocol to investigate the angiogenic effects of shock wave therapy in practical applications. Our findings suggest that ESWT increases the number of vessels in CAM. In the final biological experiment (*n* = 16 CAM per group), ESWT increased vessel number by approximately 14.3% (*p* = 0.050) and branching points by approximately 13.5% (*p* = 0.070), while mean vessel thickness decreased by approximately 8.0% (*p* = 0.047); total vascular area per image remained unchanged (*p* = 0.760). These effects were concentrated in short-to-medium, thinner-caliber vessels rather than reflecting a uniform increase in vascularity, consistent with the emergence of a denser microvascular network. This new experimental setup paves the way for future research into the angiogenic effects of ESWT and provides a multifaceted model as an intermediate step between in vitro mechanistic studies and clinical translation.

## 1. Introduction

Angiogenesis, the formation of new blood vessels from pre-existing vasculature, is crucial for maintaining and restoring the blood supply to tissues. This process plays a key role in tissue regeneration, wound healing, and the management of ischemic diseases. Extracorporeal shock wave therapy (ESWT) has evolved from its original application in urological lithotripsy into a versatile biophysical therapeutic modality for a wide range of conditions, including bone fractures and tissue regeneration in chronic wounds [1,2,3,4]. Increasing evidence indicates that ESWT promotes angiogenesis and tissue revascularization [4,5,6]. The molecular mechanisms of ESWT are mediated by acoustic waves, which transport energy through tissue and induce mechanotransduction pathways that release and activate various mediators and cytokines [3].

ESWT has been shown to activate mechanosensors such as caveolin-1 and β1-integrin on endothelial cell membranes, thereby promoting the expression of vascular endothelial growth factor (VEGF) and endothelial nitric oxide synthase (eNOS) [5,6,7].

To better understand these pro-angiogenic effects, robust and standardized experimental models are required. The chick egg chorioallantoic membrane (CAM) assay is a widely used and valuable in vivo research model for studying angiogenesis.

CAM, found in fertilized and incubated chicken eggs, is a highly vascularized extraembryonic membrane responsible for gas, metabolite, and mineral exchange in the developing chick embryo [8,9]. The superficial vascular network enables direct visualization and quantification of vascular responses to biophysical therapies, and allows for rapid assessment [9,10,11]. Previous work has demonstrated the feasibility of using the CAM model to investigate ESWT. Heimes et al. investigated the effects of applying ESWT to a collagen matrix on the surface of the chorioallantoic membrane to study its impact on the immune and angiogenic response to the transplant [12]. Wang et al. observed increased vascular growth when a culture medium containing shock wave-treated osteoblasts was applied to CAM [13]. However, a standardized protocol for the direct application of ESWT to the CAM, enabling precise control of shock-wave parameters and reproducible assessment of vascular responses, has not yet been established.

The purpose of this study was to develop a standardized experimental setup for applying ESWT to CAM to investigate its effects on blood vessel formation. We hypothesized that a standardized, direct ESWT-CAM application protocol would elicit a measurable pro-angiogenic response in the CAM vascular network. To achieve this, we followed a three-step process. First, we virtually simulated different methods of applying shock waves to CAM. Next, we conducted preliminary studies to assess the feasibility of these experimental setups. Finally, we used the most promising CAM-ESWT protocol to evaluate the angiogenic effects of ESWT in experimental studies.

## 2. Materials and Methods

**Figure 1:**
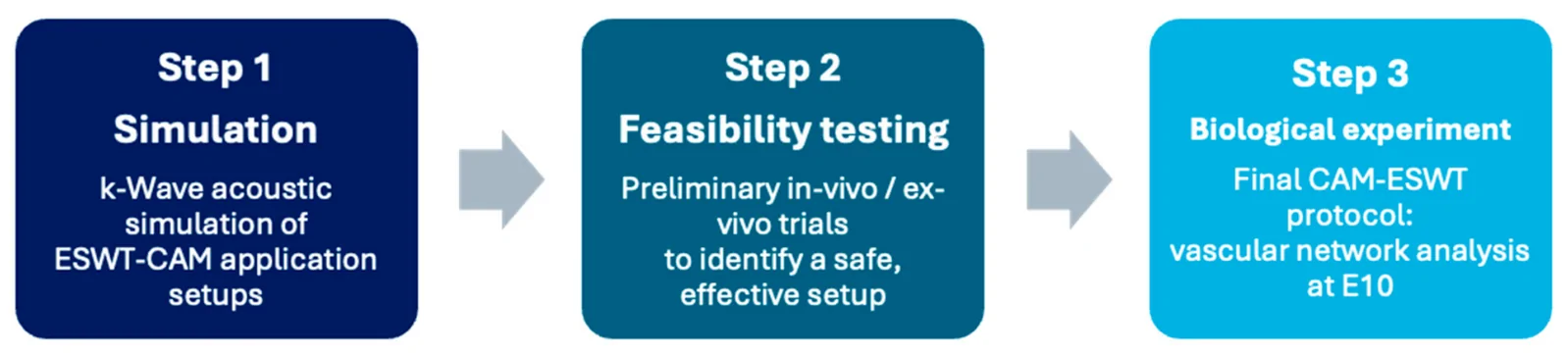
Overview of the three-step experimental workflow used to develop and apply the CAM-ESWT protocol: (1) acoustic simulation of candidate ESWT-CAM application setups (Section 3.1), (2) preliminary feasibility testing of these setups (Section 3.2), and (3) the final biological experiment assessing the angiogenic effects of ESWT on the CAM vascular network (Section 3.3 and Section 3.4).

To develop a standardized protocol for applying ESWT to CAM, we followed a three-step process depicted in Figure 1. First, we virtually simulated different methods of applying ESWT to CAM. Subsequently, preliminary studies were conducted to test various setups for practical implementation. Based on these findings, the most effective setup was employed in a final study to investigate the angiogenic effects of ESWT.

### 2.1. Ex Ovo Chorioallantoic Membrane Assay

To ensure direct access to and visualization of the CAM, an ex ovo incubation method was employed, following the protocol of Naik et al. [14]. Fertilized white Lohmann chicken eggs (LSL-hatching eggs, Schropper GmbH, Gloggnitz, Austria) were incubated horizontally at 37.6 °C and 60% relative humidity with automatic rotation (Easy 100, J. Hemel Brutgeräte, Verl, Germany). The incubator and all working surfaces were disinfected prior to use to minimize the risk of infection in the immunodeficient embryos. On embryonic day 5 (E5), all eggs were candled to confirm viability before being cracked under sterile conditions and transferred into sterile disposable weighing boats (611-0094, VWR^®^, Vienna, Austria), which were covered with a lid (D210-16, Saint-Mathieu-de-Beloeil, QC, Canada) and returned to the incubator without rotation. The vascular network of CAM develops at embryonic day 4–5 (E4–E5) and grows rapidly until E10 [15,16]. ESWT was applied to the treatment group on E5 as described in Section 2.3. On embryonic day 10 (E10), CAMs of surviving embryos were imaged as described in Section 2.4, after which embryos were euthanized by decapitation, immediately following imaging, at a developmental stage preceding the suggested onset of nociceptive processing in the chick embryo. The experimental timeline is summarized in Section 3.3. Ethics committee approval was not required, as chick embryos prior to hatching are not classified as protected animals [8,9]. Research indicates that chick embryos do not experience pain until days 13 to 14 of development, making them preferable to sentient animal models [8,9].

For each of the three candidate CAM-ESWT setups, *n* = 10 eggs were used in the preliminary feasibility experiments described in Section 3.2. For the final biological experiment, 64 fertilized eggs were cracked and transferred to the ex ovo setup, limited by incubator capacity, and allocated to the control (*n* = 32) and ESWT (*n* = 32) groups. Of these, 32 CAM (16 control, 16 ESWT) survived to E10 and were included in the analysis (four images per CAM, 64 images per group), corresponding to a survival rate of approximately 50%, which is within the expected range reported for ex ovo cultivation [17]. Survival did not differ between groups, suggesting no losses attributable to the ESWT treatment itself.

### 2.2. Simulation of ESWT-CAM Setups

The sole and direct application of Extracorporeal Shock Wave Therapy (ESWT) on the vascular network of the chorioallantoic membrane (CAM) has not been described yet. Therefore, we initially simulated different ESWT application setups to the CAM using the Matlab (2023b, Natick, MA, USA) toolbox k-wave (Version 1.4) [18,19]. The goal was to determine the pressure and energy that reach the CAM vasculature. We reconstructed the shockwave signal of the ESWT applicator using pressure measurements obtained with a needle hydrophone (Müller Instruments, Oberursel, Germany) at the respective energy level.

The acoustic properties required for the simulation (density, speed of sound, and absorption coefficient) are summarized in Table A1 in Appendix A.

An ellipsoid with two radii of 28 mm and 22 mm was used to approximate a medium-sized egg, resulting in a total volume of 54.6 mL. The amount of air inside the egg ranges from 1.5% to 8% of the egg’s volume, with 5% chosen [20].

**Figure 2:**
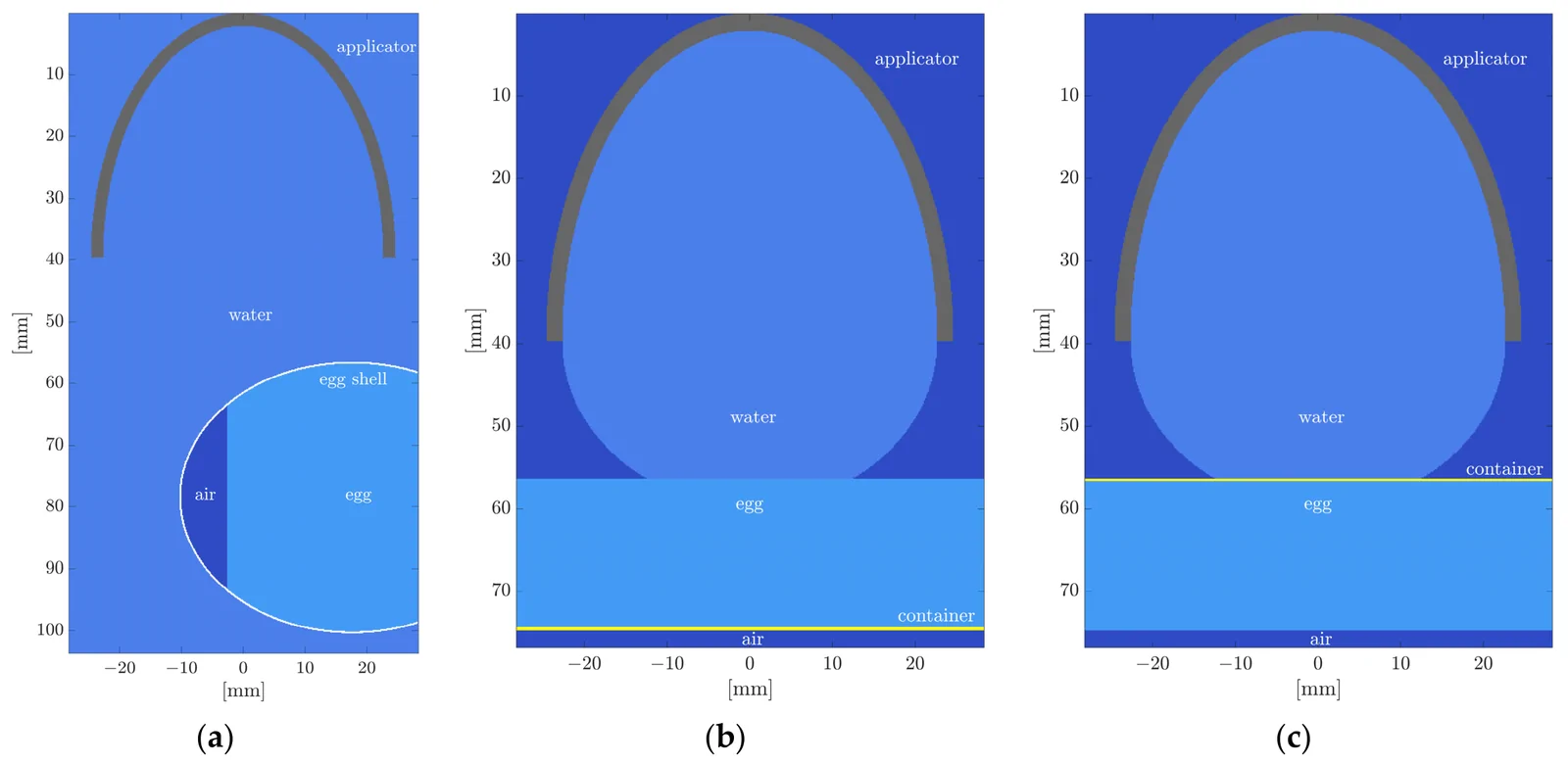
The three investigated setups, all visualized with the applicator on top to facilitate comparison of the pressure fields: (**a**) intact egg in the water bath, aiming at the region of the CAM (rotated 90° to the right during application); (**b**) ex ovo application of ESWT through a NaCl solution layer on the surface of the CAM; and (**c**) ex ovo application of ESWT through the bottom of the weighing boats (inverted in practical use, as described in Section 3.3).

Three different setups were investigated in total (see Figure 2): one setup with the intact egg in a water bath, and two ex ovo setups, one with the applicator directly positioned onto the CAM through a thin layer of 0.9% NaCl solution, and the other through the bottom of the plastic weighing boats.

For the simulation, the CUDA code provided by k-wave was used, running on a workstation equipped with an Nvidia RTX 4090 with 512 GB of RAM and an Intel Xeon Gold 6326. With a resolution of 0.14 mm, a maximum frequency of 1.225 MHz was supported.

### 2.3. Extracorporeal Shock Wave Therapy

We used the OrthoGold 100™ system (MTS Medical UG, Konstanz, Germany) with a focused applicator OE50. Practical tests were conducted using 400 shock waves at energy flux densities of 0.24, 0.17, and 0.07 mJ/mm^2^ and a frequency of 3 Hz. In the final study protocol, we ensured identical conditions on all groups, differentiating only in the ESWT administration, to mitigate confounding factors and data bias.

The CAMs of the control group were removed from the incubator for the same duration as the ESWT treatment, so that neither the accompanying drop in temperature and humidity nor the handling-related stress from movement and light exposure during the ESWT procedure would differentially affect the results; the transparent plastic lids remained in place on both groups throughout. Apart from the ESWT application itself, delivered through the outside of the plastic weighing boats, conditions were therefore identical between groups. We deliberately did not perform a sham ESWT procedure, because no comparable sham exists for this direct CAM-ESWT application and even an ESWT-treatment with a very low energy flux density might show effects on angiogenesis [21]. We therefore kept conditions as consistent as possible between groups with the sole intended exception of the ESWT application, which was applied exclusively through the plastic base of the weighing boats, leaving the CAM assay itself untouched in both groups.

### 2.4. Imaging and Image Analysis

For the final study, four randomized images of independent regions of the vascular networks of the CAMs of surviving embryos from both groups were acquired at E10 to investigate the angiogenic effects of ESWT. The imaging was performed using a light microscope (Leica M651, Leica Microsystems, Wetzlar, Germany) at 10× magnification and an SLR camera (NIKON D5600, Nikon Corp., Tokyo, Japan). Images were analyzed automatically using the deep learning-based IKOSA^®^ CAM Assay Application (KML Vision, Graz, Austria) to ensure reproducible, standardized, and observer-independent data acquisition [22,23,24]. This application uses image segmentation to quantify the number of branching points, mean thickness, and the total area and length of the vascular network [23]. Furthermore, the application allows for analysis of the thickness and length of each individual vessel. By evaluating multiple parameters, a more precise conclusion about angiogenic effects can be drawn, and distortion due to vasodilation or vasoconstriction can be avoided [10].

### 2.5. Statistical Analysis

Analyses were performed in RStudio (Version 2026.05, Posit Software, Boston, MA, USA; glmmTMB/lme4 package). Vascular parameters were modeled at the image level using generalized linear mixed models (GLMMs) with a random intercept per egg, applying a negative binomial distribution (log link) for count measures and a Gamma distribution (log link) for size measures; effects are given as percentage changes with 95% confidence intervals. Group distributions were compared with Kolmogorov–Smirnov tests and quartile composition with Pearson’s χ^2^, and quartile-wise GLMM effects were Holm-corrected. The significance threshold was *p* < 0.05.

## 3. Results

### 3.1. Simulation of Different Application Methods of ESWT on CAM

To assess the shock-wave energy at the vascular network of the chorioallantoic membrane, we simulated different ESWT applications on CAM in ovo and ex ovo. The in silico simulation provides an estimate of the expected pressure distribution in the treatment zone; direct measurements in the experimental setups are difficult to obtain and are only locally representative.

**Figure 3:**
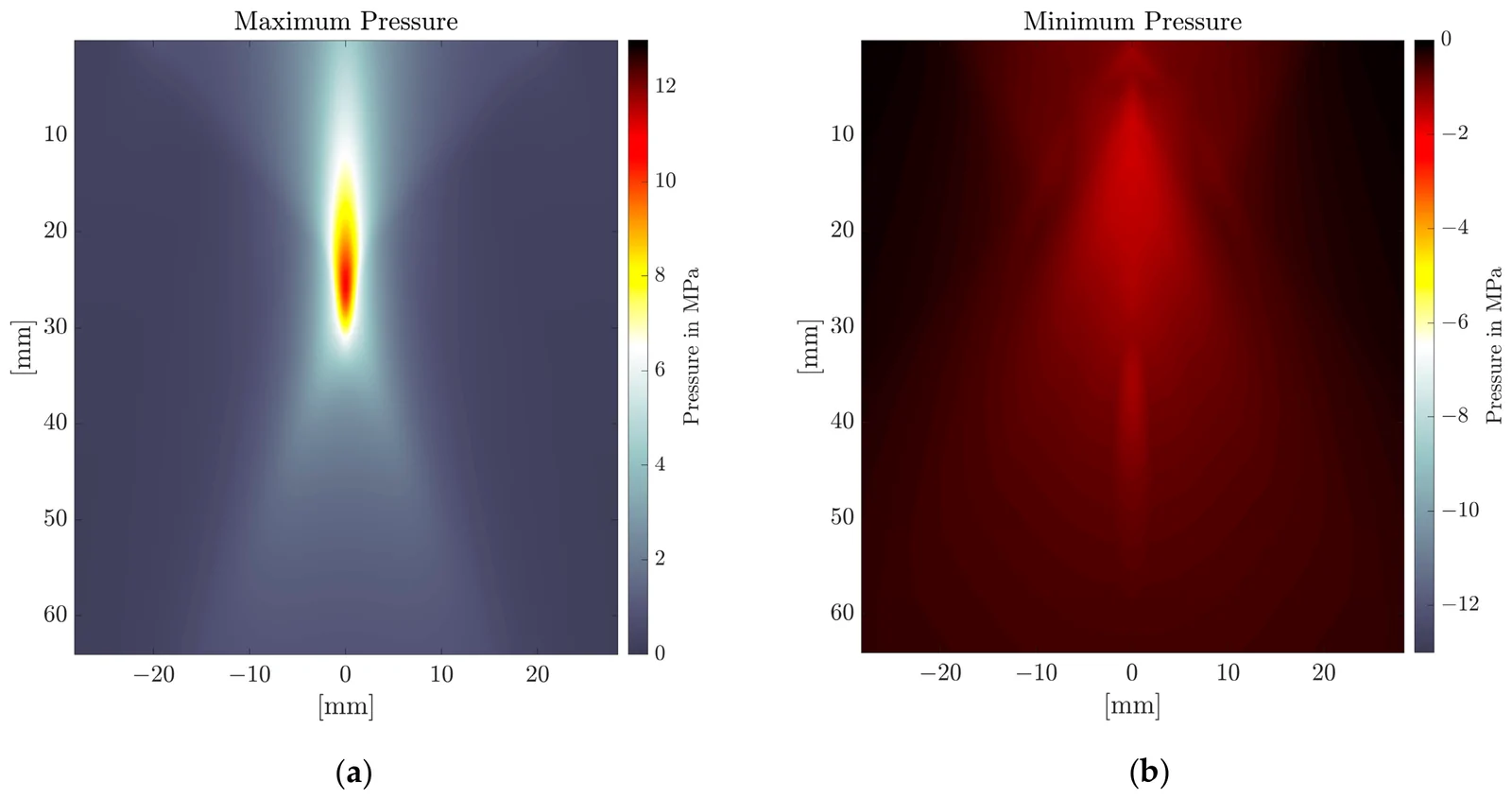
Simulation of the applicator in an empty water bath—the pressure distribution is similar to a clinical application in soft tissue. Section of the (**a**) maximum compressive and (**b**) minimum tensile pressure.

To begin with, the shockwave in an empty water bath has to be observed. This serves as a baseline for estimating how much of the original energy and pressure reaches the area of interest. In Figure 3, the pressure distributions in the focal area for the minimum and the maximum pressure are shown. Using the measurements recorded along the propagation axis at an energy setting of 0.17 mJ/mm^2^, the focal distance was adapted to 26 mm, with an average peak pressure of 10.5 MPa—serving as a basis for the water bath setup.

#### 3.1.1. Simulation: In Ovo Application of ESWT on CAM in a Water Bath

In the first simulation, we examined the study setup for ESWT application to the CAM in ovo before cracking in a water bath, following the method described by Slezak et al. [25].

**Figure 4:**
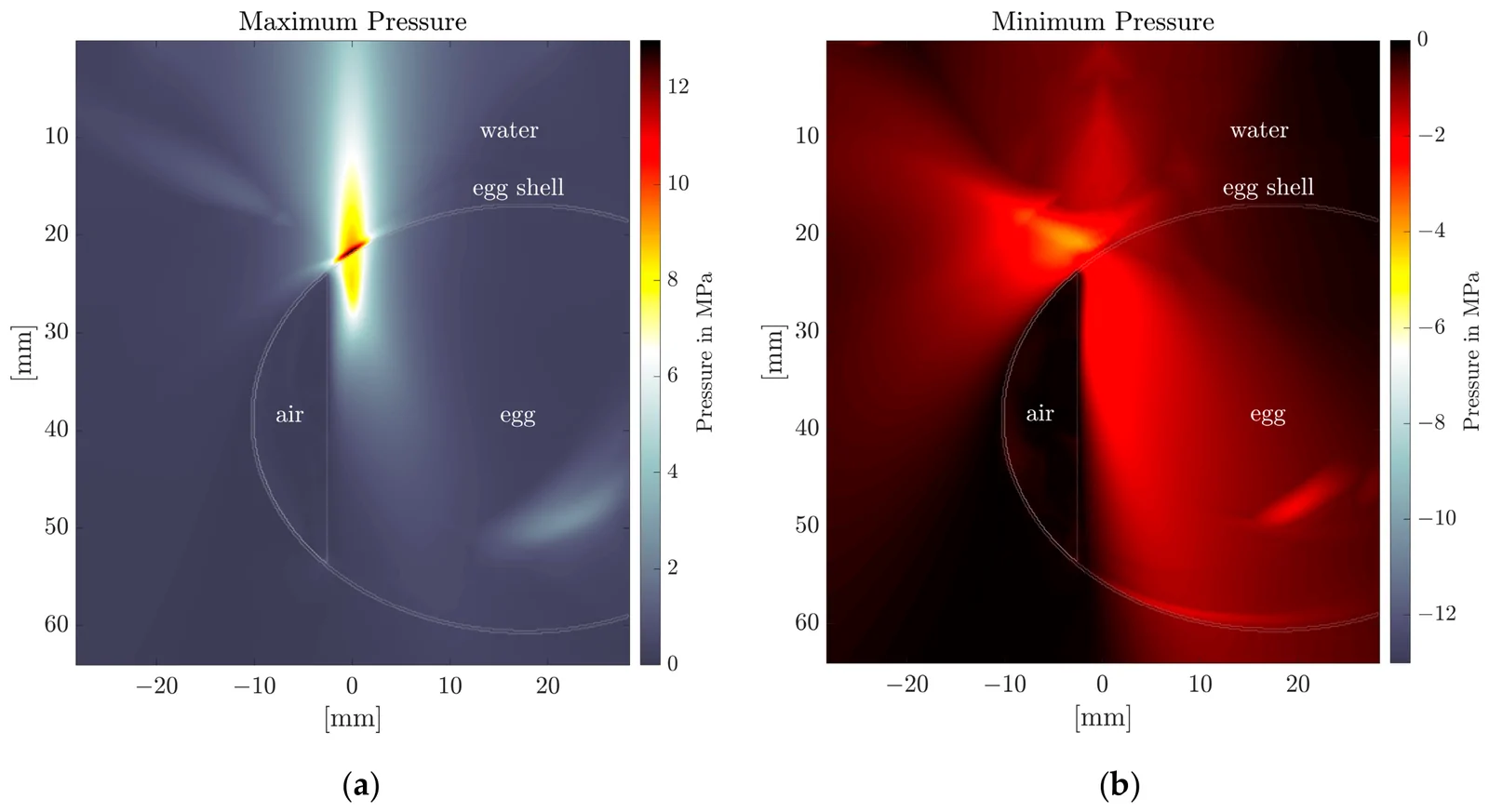
Simulation of the applicator with the complete egg introduced in the water bath, targeting the air cell of the egg, where the CAM is located. Section of the (**a**) maximum and (**b**) minimum pressure.

The focus of the ESWT was positioned at the air cell of the egg, where the CAM is located (Figure 4). Because the simulation does not account for shear waves, it may exhibit higher computational error; however, in bone at incident angles below 34° to the surface normal vector, this error was deemed not significant [26].

Reflections are clearly observable as the shockwave propagates through the egg shell, although, as anticipated, due to the thickness of the shell, only little energy is absorbed. Overall, a pressure reduction of around 15% should be expected, and a significant amount of energy reaches the center of the egg.

#### 3.1.2. Simulation: Ex Ovo Application of ESWT Through a Water Layer on the Surface of CAM

Next, we simulated the application of ESWT to the CAM surface through a thin layer of 0.9% NaCl solution in the ex ovo setup.

**Figure 5:**
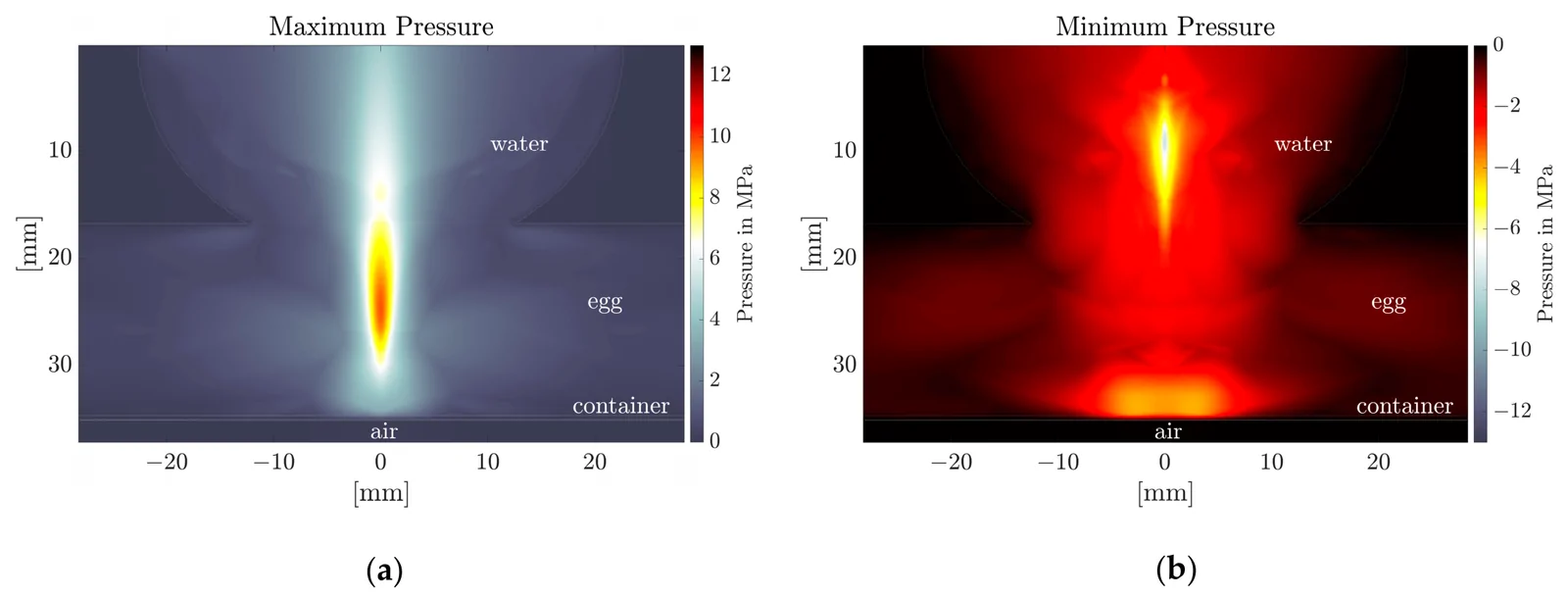
Simulation of the ex ovo application of ESWT through a water layer on the surface of CAM. Section of the (**a**) maximum and (**b**) minimum pressure.

In contrast to the water bath setup, the egg cannot be placed at arbitrary distances but is constrained by the maximum membrane height, which was measured at 22 mm. Additionally, an air interface can be found directly in the pathway of the shockwave, resulting in reflections that invert the pressure signal, mainly visible in the minimum-pressure plot (Figure 5). Directly at the air interface, an increase in tensile pressure can be observed, resulting in multiple small pressure oscillations in the shockwave signal. These effects may be larger due to the limitation of the simulation; the reflection at the air interface is underestimated by around 9%—however, the focus point of the reflections lies inside the applicator bubble.

#### 3.1.3. Simulation: Ex Ovo Application of ESWT Through the Bottom of the Weighing Boats

**Figure 6:**
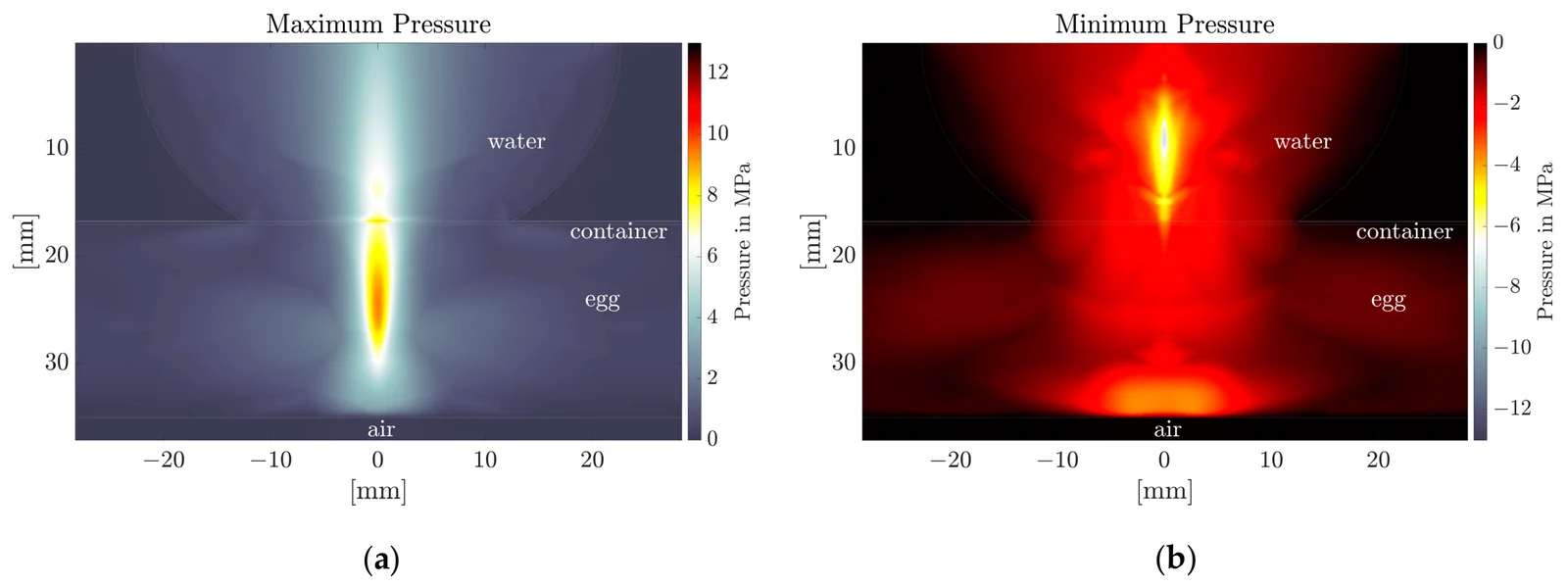
Simulation of the ex ovo application of ESWT through the bottom of the weighing boats. Instead of flipping the whole simulation upside down, only the sequence of the materials was changed. Section of the (**a**) maximum and (**b**) minimum pressure.

Finally, we conducted a simulation of ESWT application to CAM via the bottom of the weighing boats in the ex ovo setup. In practice, this setup is inverted. This representation was adopted to facilitate comparison with other setups. Like the previous simulation in Section 3.1.2, a reflection at the air interface can be seen in Figure 6, also resulting in oscillations in the pressure signal.

The plastic bottom of the weighing boats may absorb some energy, but introducing a polystyrene layer between the egg and the applicator yields only minimal changes. Even with this limitation, the deposited energy is still expected to be similar, if not greater than, that of the intact egg placed in the water bath setup.

### 3.2. Feasibility Testing of the CAM-ESWT Setups

The setups are described in Section 3.1. were tested for practical implementation to determine the most suitable research model for investigating the angiogenic effects of ESWT in CAM.

#### 3.2.1. In Ovo Application of ESWT on CAM in a Water Bath

**Figure 7:**
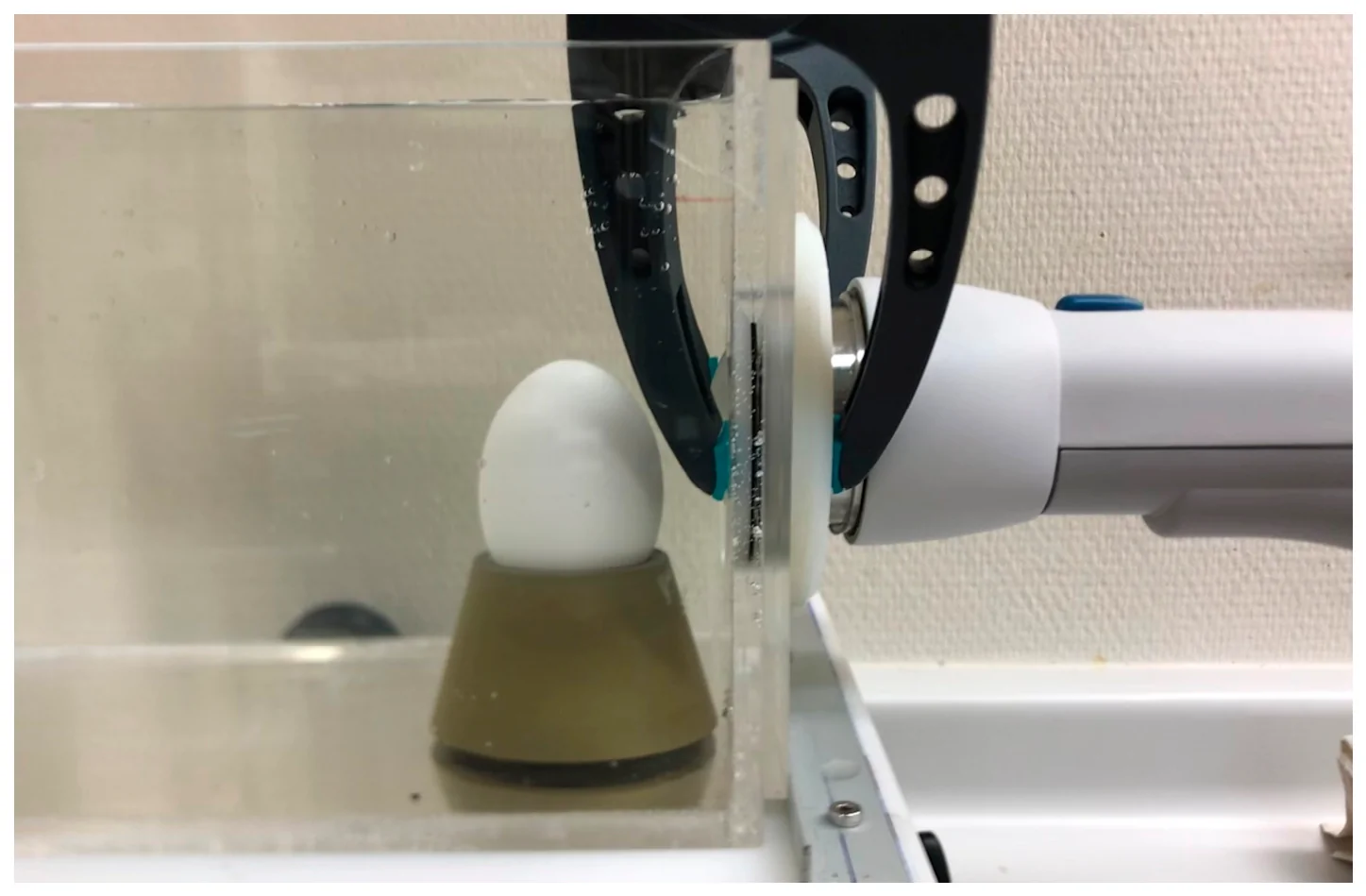
Application of ESWT on incubated eggs in the water bath.

Initially, we treated unincubated eggs with ESWT in a water bath to reduce the number of chicken embryos, in accordance with the principles of the 3Rs for the use of animals in research [27]. Figure 7 illustrates the experimental setup used to apply ESWT to the chicken eggs. The eggshells were found to break at an energy flux density of 0.24 mJ/mm^2^, as speculated in the simulation in Section 3.1.1. Based on the results of the preliminary simulation, we decided not to conduct any further tests with incubated eggs in the water bath, as it cannot be ensured that the focal zone of shock waves lies within the location of the CAM and that sufficient energy can be applied.

#### 3.2.2. Ex Ovo Application of ESWT Through a Water Layer on the Surface of CAM

Next, we applied ESWT to the CAM surface in the ex ovo setup at E5. The application was performed using a thin layer of 0.9% NaCl solution on the CAM surface to prevent damage to the vulnerable membrane. After ESWT, the fluid was aspirated to avoid impeding gas exchange. However, we observed that, despite careful handling, most CAMs were damaged. Moreover, the treatment proved to be fatal for the chicken embryos as the fluid could not be entirely aspirated. Therefore, standardization cannot be ensured due to confounding factors of the physical effects associated with the application.

#### 3.2.3. Ex Ovo Application of ESWT Through the Bottom of the Weighing Boats

**Figure 8:**
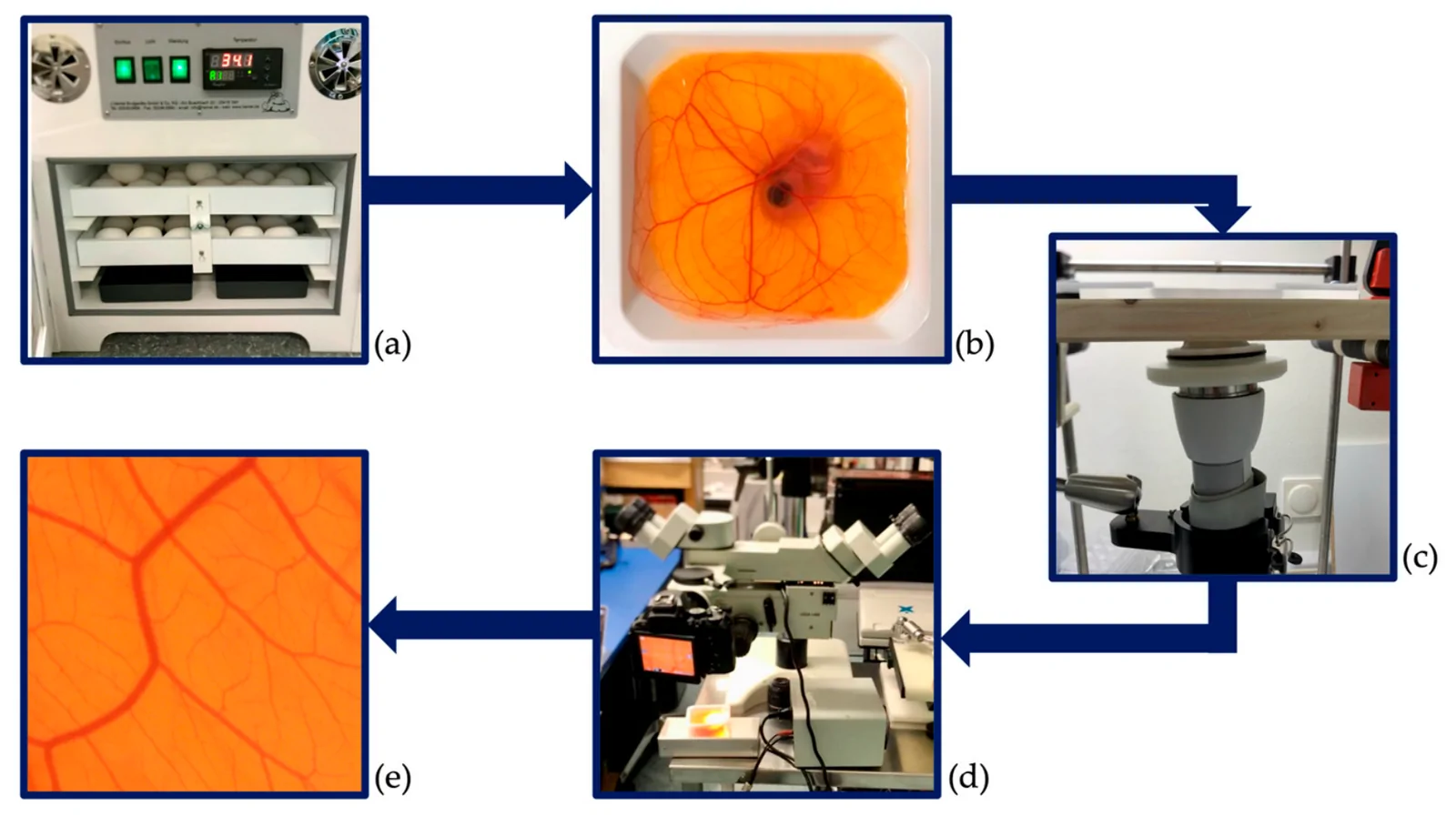
CAM-ESWT research protocol. (**a**) Start of incubation E0. (**b**) Cracking of the eggs and transfer into the ex ovo setup on E5. (**c**) Extracorporeal shock wave therapy through the bottom of the weighing boats on E5. (**d**) Imaging of the vascular network on E10. (**e**) Final image for analysis.

Finally, we tested the application of ESWT through the bottom of the weighing boats after cracking the eggs on E5 in the ex ovo setup, as depicted in Figure 8c. To ensure the transmission of the shock waves, we used contact gel between the bases of the weighing boats and the shock wave applicator, as used in clinical practice. We observed that when the energy flux densities were set at 0.24 and 0.17 mJ/mm^2^, small hemorrhages of the capillary sprouts occurred. Considering the simulation, this may be due to the reflections at the air interface, leading to negative pressure, as described in Section 3.1.3. Based on our preliminary studies, we found that delivering 400 shock waves at an energy flux density of 0.07 mJ/mm^2^ and a frequency of 3 Hz is a suitable and effective method for applying ESWT to the CAM in this setup, which lies within the low-energy range associated with pro-angiogenic and regenerative effects [28]. Our findings suggest that this method has no adverse effect on embryo survival.

### 3.3. Final CAM-ESWT Protocol

By conducting simulations and practical investigations, we have established an effective research setup to investigate the effects of ESWT on angiogenesis in CAM. The standardized application protocol, as illustrated in Figure 8c, was subsequently implemented to comprehensively assess vascular responses.

### 3.4. Results of the Analysis of the Vascular Network of the CAM

In the final step of our study, we evaluated the effects of ESWT on angiogenesis in CAM at E10. Control and ESWT groups (16 samples each, 64 images per group) yielded 300,431 vessels analyzed via IKOSA CAM Assay, with all inferential comparisons performed at image level using GLMMs (Gamma error with log-link for the median size measures, negative binomial with log-link for the count measures)—Table 1 provides a descriptive overview of the two groups.

ESWT increases vessel count by approximately 14.3% (*p* = 0.050, 95% CI 1.00–1.31), and total branching points rise by approximately 13.5% (*p* = 0.070, 95% CI 0.99–1.30). At the same time, treated vessels are thinner (−8.0%, *p* = 0.047, 95% CI 0.85–1.00), while median length shows a non-significant reduction (−5.3%, *p* = 0.143, 95% CI 0.88–1.02).

**Figure 9:**
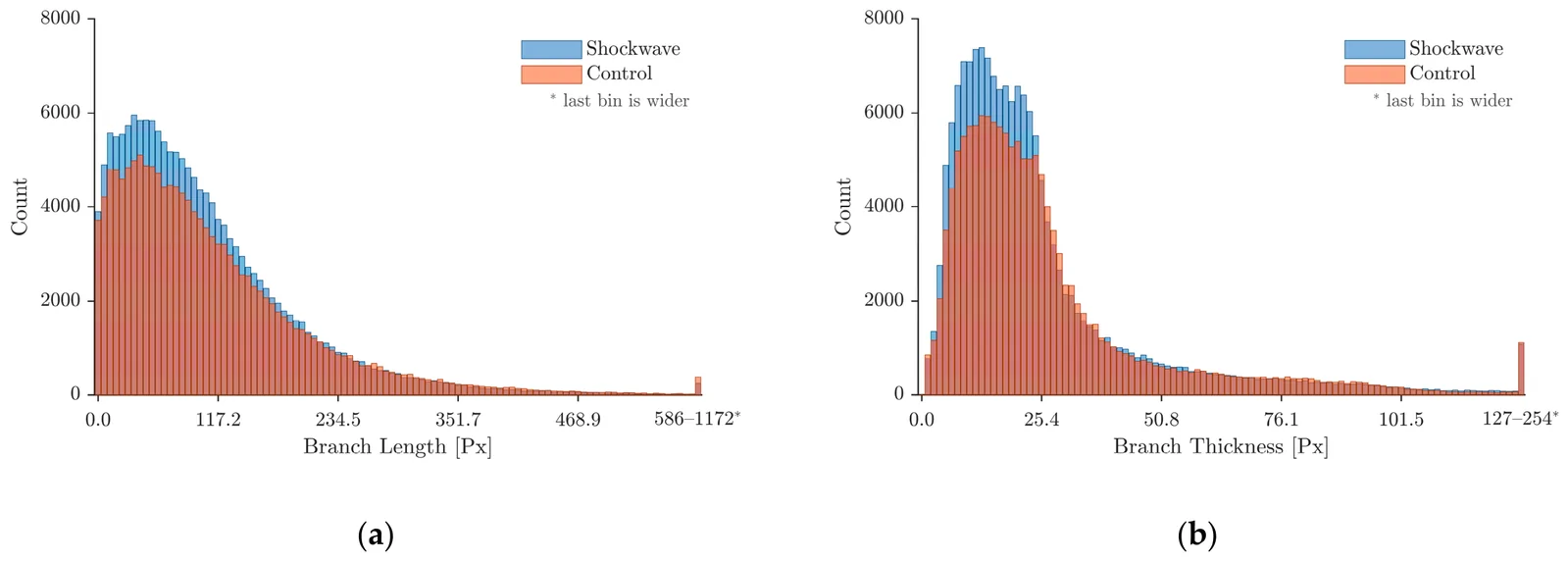
Histogram showing the distribution of the blood vessels for length (**a**) and thickness (**b**) across the control and the ESWT group. Bin widths are 5.86 px and 1.27 px, respectively (with the last bin representing a wider range for visual clarity).

These shifts in central tendency can be explained by a shift in the distributions between groups, as shown in Figure 9. The Kolmogorov–Smirnov tests reject distributional equality for both length (D = 0.027) and thickness (D = 0.054, both *p* ≪ 0.001), and Pearson χ^2^ on quartiles confirms a shift in proportional composition (length χ^2^ = 281.2; thickness χ^2^ = 725.6; both *p* ≪ 0.001). For length, this manifests as an underrepresentation of Q4 with a surplus across Q1–Q3, whereas for thickness it follows a monotonic gradient, with Q1 and Q2 overrepresented and Q3 and Q4 underrepresented. Per quartile, GLMMs show a monotonic gradient in absolute changes for thickness (Q1 + 34%, Q2 + 22%, Q3 + 9%, Q4 − 1%) and a plateau across the smaller to medium length classes (Q1 + 18%, Q2 + 21%, Q3 + 18%, Q4 + 2%). After Holm correction, no individual quartile reaches significance, but the pattern is internally consistent, with the surplus concentrated in small to medium vessels and no change in the largest. Despite the higher vessel count, total vessel area per image is unchanged (*p* = 0.760, 95% CI 0.93–1.06).

## 4. Discussion

In the present study, we developed a standardized application protocol to investigate the effects of ESWT on angiogenesis in the CAM assay. Using a three-step process of simulation, feasibility testing, and experimental application, we identified ex ovo delivery through the bottom of the weighing boats as the most suitable setup.

The simulations for the in ovo application of ESWT on CAM in a water bath predicted two limitations: First, without modifying the applicator, the device’s membrane would limit penetration depth due to its minimal height of 15 mm. Second, the integrity of the eggshell may be compromised by the shock wave, considering that Smith et al. reported a minimum pressure of 5.3–7.6 MPa for artificial kidney stone comminution [29]. Feasibility testing of this setup confirmed that the eggshell is prone to cracking under high pressure. For the ex ovo setups, pressure profiles and signals differed only slightly, but application through the bottom of the weighing boats was more favorable for embryo survival in preliminary practical testing. Although reflections at the air interface produce oscillations with an increased tensile component, the main negative reflection focus lies outside the egg. These additional tensile components could enhance ESWT efficacy through cavitation, but could equally cause undesirable side effects such as cell or tissue damage, which warrants further investigation.

Analyzing the vascular response to ESWT treatment, an increase in vessel number was observed (*p* = 0.050), while the increase in branching points did not reach statistical significance (*p* = 0.070); a larger cohort will be needed to confirm these trends. Our findings are in line with Heimes et al. [12], who reported pro-angiogenic effects of ESWT applied to a collagen matrix implanted on the CAM. In contrast to that transplant-based approach, our setup delivers ESWT directly to the native CAM vasculature, allowing the vascular response to be assessed without an intermediary matrix.

The quartile-resolved analysis shows a monotonic gradient in thickness changes (Q1 + 34%, Q2 + 22%, Q3 + 9%, Q4 − 1%), indicating that the surplus vessels are concentrated in the smallest-caliber classes while the largest vessels remain essentially unaffected. This composition shift is compatible with the formation of new small vessels through two non-exclusive mechanisms. Sprouting angiogenesis generates new thin endothelial outgrowths, whereas intussusceptive angiogenesis divides pre-existing vessels through transluminal tissue pillars into shorter and thinner daughter segments [16,30]. Vasoconstriction would fit the observed thickness gradient, but would predict a more uniform shift in vessel length than the one observed, and is therefore unlikely to be the dominant driver. A vascular steal phenomenon, in which flow is diverted away from existing beds, would instead be expected to reduce pre-existing small-caliber vessels [31]. The length distribution is centered on Q2 and Q3 rather than solely Q1, suggesting that the response may have begun before E10 and that the new vessels had already started to elongate. The CAM at E5–E10 is in an active phase of vascular expansion, in which sprouting angiogenesis dominates capillary network growth from E5 to E7 while intussusceptive growth prevails from E8 to E12 [16]. The observed increase in thinner vessels with preserved large-vessel architecture is therefore consistent with ESWT augmenting both the physiological sprouting process and the subsequent intussusceptive expansion phase. These mechanistic interpretations remain inferential, as two-dimensional vessel morphometry cannot directly distinguish sprouting from intussusception or measure functional integration into a mature vascular network.

Several molecular techniques are well suited to validate the signaling pathways presumed to mediate ESWT-induced angiogenesis in the CAM model in future work. Quantitative real-time PCR (qPCR) is well established in CAM research and could quantify transcript-level changes in VEGF, VEGF-A, FGF-2, HIF-1α, and eNOS [7,16,32,33,34,35]. The Ang-1/Ang-2 ratio would be of particular interest to assess vascular maturation [36,37]. At the protein level, Western blot analysis could confirm translational changes in VEGF, eNOS, and phosphorylated Erk1/2 and Akt, key downstream effectors of ESWT-induced mechanotransduction via caveolin-1 and β1-integrin and immunohistochemistry could spatially localize these factors within the CAM tissue layers to correlate molecular expression with the morphological vascular changes observed [7].

The energy flux density (EFD) of 0.07 mJ/mm^2^ used in the final experiment falls within the lower end of the clinically applied ESWT spectrum, but not below it: clinical soft-tissue wound protocols use EFDs of 0.037–0.1 mJ/mm^2^, musculoskeletal protocols for rotator cuff tendinopathy span 0.013–0.15 mJ/mm^2^, and bone nonunion protocols typically use higher EFDs of 0.4–0.7 mJ/mm^2^ to promote osteogenesis [38,39,40,41]. Notably, 0.07 mJ/mm^2^ lies within the range specifically associated with pro-angiogenic effects: shock waves at 0.03 mJ/mm^2^ have been shown to upregulate VEGF and eNOS via mechanosensor activation, EFDs as low as 0.012–0.045 mJ/mm^2^ activate mechanosensory complexes in endothelial cells, and a therapeutic window for myocardial regeneration has been defined in which angiogenic gene expression and endothelial proliferation increase dose-dependently from 0.01 to 0.15 mJ/mm^2^, with no cellular damage below 0.27 mJ/mm^2^ [7,21,28]. We acknowledge that the selection of 0.07 mJ/mm^2^ partly reflects a compromise imposed by the fragility of the CAM model: preliminary experiments showed that higher energy densities (0.17 and 0.24 mJ/mm^2^) caused hemorrhage and embryo death, consistent with reports that endothelial progenitor cells shift from proliferation to apoptosis at 0.16 mJ/mm^2^ and that a cellular damage threshold of around 0.27 mJ/mm^2^ exists for human endothelial cells [28,42].

A further limitation is that we evaluated only a single ESWT dose delivered in a single treatment session. This was a deliberate methodological choice to first establish a standardized delivery protocol. A single in vivo exposure can nonetheless yield a durable angiogenic response [43]. Increasing the EFD in a single session further risks crossing the reported endothelial damage threshold of approximately 0.27 mJ/mm^2^, whereas the effect of multiple, lower-energy treatments could not be tested within the short, ethically constrained CAM observation window (E5–E13) and remains an important direction for future work.

The CAM, with its immature vasculature and lack of surrounding protective tissue, is likely more susceptible to mechanical damage than adult tissues in clinical settings, possibly limiting the ESWT-CAM protocol. These distinctive features of CAM may alter the vascular response to mechanical stimuli, so quantitative effect sizes observed here should not be extrapolated directly to human tissue without further validation in mammalian models.

## 5. Conclusions

The present study introduces a CAM setup specifically designed to investigate the angiogenic effects of ESWT under controlled experimental conditions. It offers advantages, including cost-effectiveness, rapid assessment, and ethical refinement, compared with other animal models. This setup addresses key methodological challenges, including standardizing shock-wave delivery to ensure reproducible conditions and integrating multiple assessment modalities to comprehensively evaluate angiogenic responses. By combining the advantages of the CAM model with optimized ESWT application protocols, this approach offers a valuable method for identifying optimal treatment parameters and for investigating underlying molecular pathways, serving as an intermediate step between in vitro mechanistic studies and translation into targeted clinical applications.

## Figures and Tables

**Table 1:** Descriptive statistics of the blood vessels per group. Total length of the vascular network, number of vessels (N), mean and median length, mean and median thickness (all in pixels), and the total number of branching points (BP).

Group	N	Total Length [px]	Total Area [px^2^]	Mean Length [px]	Median Length [px]	Mean Thickness [px]	Median Thickness [px]	Total BP
Control	141,051	16 × 10^6^	436 × 10^6^	113.5	89.97	27.4	20.82	70,863
ESWT	159,380	17 × 10^6^	431 × 10^6^	106.9	86.43	25.7	19.38	79,519

## Data Availability

The data presented in this study are available on request from the corresponding author. The data are not publicly available due to the ongoing industrial development of the CAM application software (V39).

## Appendix A

The acoustic properties required for the simulation (density, speed of sound, and absorption coefficient) are summarized in Table A1. Values for water, air, and polystyrene were taken from the literature [44,45,46]; egg content properties were adapted from [47], with the absorption coefficient assumed to be similar to blood [48], and eggshell properties were derived from the elastic modulus [49], with the absorption coefficient assumed to be similar to human skull bone [50]. Given the eggshell and polystyrene thicknesses of only 0.3 mm each, their influence on the overall result is expected to be limited.

**Table A1:** Material Properties used for the simulation.

Material	Density (kg/m^3^)	Speed of Sound (m/s)	Absorption Coefficient (dB/cm)	Source
Water	998	1481	0.00424	[46]
Air (nominal)	1.2	343	1.62	[44]
Air (simulation)	100	343	1.62	see text
Polystyrene	1050	2350	3.00	[44,45]
Egg contents	1030	1520	0.20	[47,48]
Eggshell	2241	3700	10.00	[49,50,51]

Due to the high acoustic impedance difference between water and air, which produces artifacts at lower simulation resolutions, an increased density for air (100 kg/m^3^) was used. This primarily affects reflections at material interfaces, thereby underestimating the reflective component and leading to a more rapid loss of energy in the system. The error was estimated using Snell’s law for reflection, indicating that the original values would yield a reflection of 99.9% of the energy, whereas the chosen value yields 91.1%. This difference means that the reflected energy is underestimated by approximately 9% in the simulation. The reflection is most clearly visible in Figure 5b, where the inversion at the air interface converts the compressive wave into a tensile wave. Of the two resulting focal zones, only one lies within the egg at the level of the CAM, whereas the second reflection zone is located within the applicator head itself. Consequently, the underestimated reflectivity acts predominantly on the focus inside the applicator rather than on the treatment plane, so the energy reaching the CAM is comparatively insensitive to the chosen air density. The simulations should therefore be regarded as estimates of the pressure distribution in the treatment zone rather than as absolute dosimetry.
